# An integral equation method for the homogenization of unidirectional fibre-reinforced media; antiplane elasticity and other potential problems

**DOI:** 10.1098/rspa.2017.0080

**Published:** 2017-05-10

**Authors:** Duncan Joyce, William J. Parnell, Raphaël C. Assier, I. David Abrahams

**Affiliations:** 1 School of Mathematics, University of Manchester, Oxford Road, Manchester M13 9PL, UK; 2 Isaac Newton Institute, University of Cambridge, 20 Clarkson Road, Cambridge CB3 0EH, UK

**Keywords:** homogenization, potential problem, antiplane elasticity, fibre-reinforced composite

## Abstract

In Parnell & Abrahams (2008 *Proc. R. Soc. A* **464**, 1461–1482. (doi:10.1098/rspa.2007.0254)), a homogenization scheme was developed that gave rise to explicit forms for the effective antiplane shear moduli of a periodic unidirectional fibre-reinforced medium where fibres have non-circular cross section. The explicit expressions are rational functions in the volume fraction. In that scheme, a (non-dilute) approximation was invoked to determine leading-order expressions. Agreement with existing methods was shown to be good except at very high volume fractions. Here, the theory is extended in order to determine higher-order terms in the expansion. Explicit expressions for effective properties can be derived for fibres with non-circular cross section, without recourse to numerical methods. Terms appearing in the expressions are identified as being associated with the lattice geometry of the periodic fibre distribution, fibre cross-sectional shape and host/fibre material properties. Results are derived in the context of antiplane elasticity but the analogy with the potential problem illustrates the broad applicability of the method to, e.g. thermal, electrostatic and magnetostatic problems. The efficacy of the scheme is illustrated by comparison with the well-established method of asymptotic homogenization where for fibres of general cross section, the associated cell problem must be solved by some computational scheme.

## 1. Introduction

A classical problem in the mechanics of inhomogeneous media is to attempt to replace the two-dimensional potential problem ∇⋅(*μ*(***x***)∇*w*(***x***))=0, where ***x***=(*x*_1_,*x*_2_) and *μ*(***x***) and *w*(***x***) are periodic (scalar) functions that vary rapidly with ***x***, by an equivalent problem of the form

1.1 $$\nabla \cdot (\mu^{*}\cdot \nabla w_{*}(\text{x}))=0,$$

where w_*_ is the leading-order displacement field and μ* is the second-order tensor of effective shear moduli [1]. To do this, a so-called *separation of scales* between the micro and macro lengthscales must be assumed. In Cartesian coordinates, (1.1) can be written in the indicial form

$$\frac{\partial }{\partial x_{i}}\left(\mu_{ij}^{*}\frac{\partial w_{*}}{\partial x_{j}}\right)=0,$$

where $\mu_{ij}^{*},(i,j=1,2)$ refers to the *ij*th component of the tensor in Cartesian coordinates. For orthotropic materials, for example, $\mu_{ij}^{*}=\mu_{1}^{*}\delta_{i1}\delta_{j1}+\mu_{2}^{*}\delta_{i2}\delta_{j2}$. The path to (1.1) is the process of *homogenization* and μ* depends strongly on the geometrical and physical properties of the medium in question [2,3]. Noting that the equations arise from the equilibrium equation ∇⋅***σ***=0, where ***σ***=***μ***⋅***e***, and ***e***=∇*w*, it is stressed that the problem posed has broad applicability, as summarized in table 1. To fix ideas here, the application to antiplane elasticity shall be described so that *w* is the out-of-plane displacement.

**Table 1.** The numerous application areas associated with the potential problem, together with corresponding variables. Here, attention is restricted to two-dimensional problems. It is noted that the acoustic scenario is applicable only in the dynamic setting of course and furthermore (***ρ***^−1^)_*ij*_ refers to the *ij*th component of the inverse of the acoustic density tensor.

application	*σ*_*i*_	*e*_*i*_	*w*	*μ*_*ij*_
antiplane elasticity	antiplane stress vector (*σ*_13_,*σ*_23_)	displacement gradient ∇*w*	displacement *w*	shear moduli *μ*_*ij*_
thermal conductivity	heat flux *q*_*i*_	temperature gradient −∇*T*	temperature *T*	thermal conductivity *k*_*ij*_
electrical conductivity	electrical current *J*_*i*_	electric field *E*_*i*_=∇*Φ*	electric potential *Φ*	electrical conductivity ${\bar{\sigma }}_{ij}$
dielectrics	displacement field *D*_*i*_	electric field *E*_*i*_=∇*Φ*	electric potential *Φ*	electric permittivity *ϵ*_*ij*_
magnetism	magnetic induction *B*_*i*_	magnetic field *H*_*i*_=∇*Ψ*	magnetic potential *Ψ*	magnetic permeability *μ*_*ij*_
porous media	weighted velocity *ηv*_*i*_	pressure gradient ∇*p*	pressure *p*	permeability *k*_*ij*_
diffusion	diffusion flux *j*_*i*_	concentration gradient ∇*c*	concentration *c*	diffusivity *D*_*ij*_
acoustics	acceleration ∂**v**/∂*t*	pressure gradient −∇*p*	pressure *p*	inverse density tensor (***ρ***^−1^)_*ij*_

**Figure 1.**
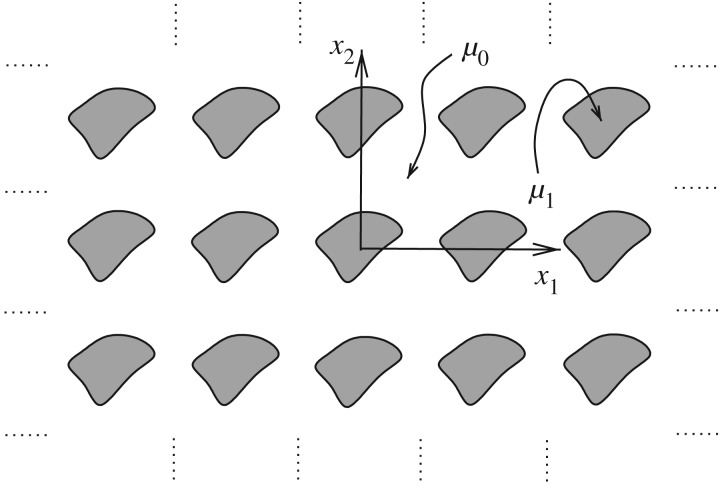
Cross section of an inhomogeneous medium with piecewise constant material property *μ*(***x***). The unidirectional fibre-reinforced composite medium depicted here has general periodic microstructure; in particular, the fibres have non-circular cross sections.

Assume now that *μ*(***x***) is piecewise constant, and a cross section of the medium takes the form as depicted in figure 1 so that the material can be classified as a unidirectional fibre-reinforced composite (FRC), so that fibres of general cross sections shall be considered. Such media are used in a multitude of applications where rather specific material properties are required in order to perform a task effectively and where naturally available homogeneous media are not effective or efficient [2]. In particular, materials of this form can provide high tensile stiffness and/or high directional conductivity while remaining relatively light by using only a small volume fraction of the fibre phase.

The microstructural lengthscales of such inhomogeneous media are becoming ever smaller with an increasing ability to engineer microstructures for improved macroscopic performance [4]. It is frequently convenient to consider the problem in the context of wave propagation so that an inertia term is added to the governing equation, and the effective medium is then governed by, for example

1.2 $$\nabla \cdot (\mu^{*}\cdot \nabla w_{*}(\text{x}))+\rho_{*}\omega^{2}w_{*}(\text{x})=0,$$

where in the context of antiplane elasticity ρ_*_ is the effective mass density and where time-harmonic motion *e*^−i*ωt*^ has been assumed, identifying *ω* as the angular frequency. By considering this dynamic context, a natural macroscopic lengthscale is introduced: the wavelength of the propagating effective wave. Intrinsic to the subject of homogenization then, where quasi-static properties are determined, is that the wavelength of the propagating wave is much longer than the microstructural lengthscale.

A number of methods have proved extremely successful at predicting μ* in both the static and quasi-static regimes, including the method of asymptotic homogenization (MAH) [1,3,5–7], the equivalent inclusion method [8,9], boundary element solutions of integral equations [10] and the use of Fourier transforms [11–13]. All schemes rely on separation of scales and periodicity—the fact that a periodic cell is representative of the entire medium. We stress, however, that general fibre cross sections shall be considered here, and the semi-analytical approach developed is able to determine explicit expressions for the effective properties with minimal computational resource. This is in contrast with existing homogenization schemes where numerical methods are required and results must be computed each time a parameter is varied, e.g. volume fraction. Having general expressions in the volume fraction is of great utility and practicality in materials design and optimization.

Moving away from the separation of scales regime, a significant amount of work has been undertaken that incorporates propagation at finite frequencies and particularly when the wavelength of the propagating wave is of the order of the microstructure, when dynamic effects become important. In this context, the microstructure can be designed to manipulate the wave-carrying capabilities of the medium. In particular for periodic materials, the microstructure can be chosen in order that the medium acts as a wave filter, for waves in certain frequency ranges (the so-called stop bands) are unable to propagate. Methods devised to determine the band gap structure are the plane wave expansion technique [14], multipole methods [15], and high-frequency homogenization [16]. See the useful review [17] for further details. Low-frequency effective properties can thus be deduced numerically from these schemes by considering propagation near the origin of the dispersion curves in question. If from the outset, however, one is interested purely in the low-frequency limit where homogenization applies, then there is no need to determine this full dynamic behaviour.

**Figure 2.**
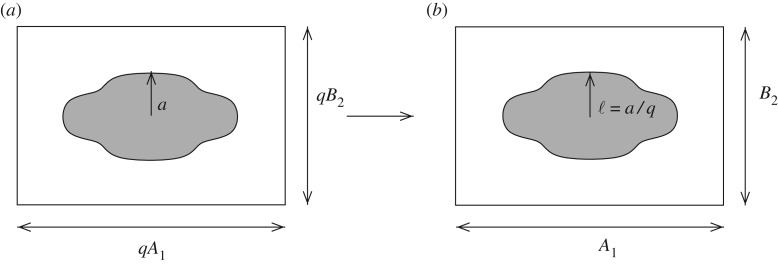
Figure depicting the dimensional (*a*) and non-dimensional (*b*) periodic cell (and associated lengthscales) in the case of rectangular periodicity and fibre with cross section such that the material is at most macroscopically orthotropic.

Here, then, a strict separation of scales shall be considered; the homogenization regime is assumed to hold and the material responds as an effective medium with uniform properties. The key novelty of the proposed scheme is the form of solutions that are derived as shall be illustrated below. The method to be discussed extends the work in [18] (referred to as PA below), where a new homogenization scheme was devised based on the integral equation form of the governing equation, considering antiplane wave propagation in the low-frequency limit, so that an equation of the form (1.2) was derived, and the leading-order result was determined. The work of PA was itself inspired by the method introduced in [19], in which expressions for the effective elastic properties of three-dimensional random particulate media were determined, but restricted to the dilute-dispersion limit [20]. Returning to the two-dimensional periodic medium considered here, although the method itself may be applied to a material of any macroscopic elastic symmetry, attention shall be restricted to microstructure that gives rise to orthotropic effective properties for clarity of exposition. In this case, $\mu_{ij}^{*}=\delta_{1i}\delta_{1j}\mu_{1}^{*}+\delta_{2i}\delta_{2j}\mu_{2}^{*},\;(i,j=1,2)$ when written with respect to the principal axes of anisotropy. This means that the fibre cross section is restricted to having two planes of reflectional symmetry as indicated in figure 2. In PA, the effective moduli were shown to take the following rational function form at leading order

1.3 $$\mu_{j}^{*}=\frac{1+C_{1j}\phi }{1+D_{1j}\phi }.$$

The coefficients $C_{1j}$ and $D_{1j}$ depend upon the shape of the fibre cross section, the periodic lattice geometry and the ratio of fibre to host shear moduli. Upon extending the method to higher orders, for *circular cylindrical fibres* the following result is derived in this work:

1.4 $$\mu_{j}^{*}=\frac{1+C_{1j}\phi +C_{4j}\phi^{4}+C_{6j}\phi^{6}+C_{7j}\phi^{7}+C_{8j}\phi^{8}+\cdots }{1+D_{1j}\phi +C_{4j}\phi^{4}+C_{6j}\phi^{6}+C_{7j}\phi^{7}+C_{8j}\phi^{8}+\cdots }$$

and additional general expressions are determined for fibres of more complex cross section. In principle, the scheme presented can be extended to three dimensions and more complex microstructural geometries.

As in PA, it shall be assumed that all fibres in the composite are identical, that all phases are isotropic and that the lattice geometry and fibre cross sections are restricted such that the effective medium appears to be, at most, orthotropic on the macroscale. In §2, the governing equations are summarized and the associated integral equations are derived. The integral equation methodology is then described in §3 and the manner in which effective properties are determined is presented in §4. Results are given in §5 for a variety of media before concluding in §6. Some of the more detailed analysis is presented in appendices in order to aid the flow of the paper.

It is reiterated that although the method is described here in the context of antiplane elasticity and expressions for the effective antiplane shear moduli $\mu_{1}^{*}$ and $\mu_{2}^{*}$ are derived, the method is equally applicable to any of the applications summarized in table 1.

## 2. Governing equations

The microstructure of the medium in question is illustrated in figure 2. Unidirectional fibres, considered so long as for the problem to be assumed two dimensional, are positioned on a periodic lattice and their cross section is taken as general with the restriction that the macroscopic anisotropy is at most orthotropic. The problem shall be formulated in Cartesian coordinates, with the *x*_3_-coordinate running parallel to the fibre axis and the *x*_1_*x*_2_-plane being the plane of periodicity. The location of the centre of the (*s*, *t*)th periodic cell is defined by the lattice vector

2.1 $$\text{R}(s,t)=q(s{\text{l}}_{1}+t{\text{l}}_{2}),\;s,t\in Z,$$

for some two-dimensional vectors ***l***_1_,***l***_2_. Attention is restricted to the case where each periodic cell contains a single fibre. The lengthscale *q* can be considered as the characteristic lengthscale of the periodic cell and therefore of the microstructure of the medium. Consider a distribution of fibres that induces macroscopic *orthotropy* so that

$${\text{l}}_{1}=(A_{1},B_{1})\;\mathrm{and}\;{\text{l}}_{2}=(0,B_{2}),\;A_{1},B_{1},B_{2}\in R^{+},$$

and the periodic cell is a parallelogram with area $q^{2}R=q^{2}A_{1}B_{2}$ (e.g. figure 2). Each cell, therefore, consists of a fibre of general cross section occupying the domain *V*_*st*_ embedded within the host phase which is denoted by *V*_0_. The shear modulus and mass density of the host (fibre) is denoted as *μ*_0_ (*μ*_*I*_) and *ρ*_0_ (*ρ*_*I*_), respectively.

The lengthscale *a* associated with the fibre cross section is introduced by defining the boundary of the cross section in terms of circular cylindrical coordinates, i.e.

2.2 r(θ)=af(θ),θ∈[0,2π],

with *f*(*θ*)≥1 and for some *θ*∈[0,2*π*], *f*(*θ*)=1. Hence, $a=\min_{\theta \in [0,2\pi ]}(r(\theta ))$ on the boundary of the fibre. This choice is convenient for calculations that will be carried out in appendix A associated with incorporating the *shape* of the fibre cross section into the analysis.

Horizontally polarized shear (SH) waves, otherwise known as antiplane waves, are considered to propagate through the medium. The associated time-harmonic elastic displacement, polarized in the *x*_3_-direction is denoted by *w*(***x***), where it is recalled that ***x***=(*x*_1_,*x*_2_). The elastic displacement *w* is governed by

2.3 $$\nabla^{2}w(\text{x})+(k_{0}^{2}+(k_{I}^{2}-k_{0}^{2})\chi (\text{x}))w(\text{x})=0,$$

where ∇=(∂/∂*x*_1_,∂/∂*x*_2_) and where *k*_0_=*ω*/*c*_0_ and *k*_*I*_=*ω*/*c*_*I*_ are the wavenumbers of the host and fibre, respectively, and where $c_{0}^{2}=\mu_{0}/\rho_{0}$ and $c_{I}^{2}=\mu_{I}/\rho_{I}$ are the squares of the phase velocities of the two phases. The so-called *indicator function* *χ*(***x***) is defined by

$$\chi (\text{x})=\left\{ \begin{array}{ll} 1 & \text{ if }\text{x}\in V_{st},\;s,t\in Z,\\ 0 & \text{if }\text{x}\in V_{0}. \end{array}\right.$$

The associated free-space Green’s function for the host domain satisfies

2.4 $$(\nabla^{2}+k_{0}^{2})G(\text{x}-\text{y})=\delta (\text{x}-\text{y})\;\mathrm{and is therefore}\;G(\text{x}-\text{y})=\frac{1}{4i}H_{0}^{\left(1\right)}(k_{0}|\text{x}-\text{y}|),$$

where $H_{0}^{\left(1\right)}(r)$ denotes the Hankel function of the first kind and zeroth order.

Combining (2.3) and (2.4) appropriately, imposing boundary conditions of continuity of displacement and traction on fibre/host interfaces, the problem is restated in integral equation form as

$$w(\text{x})=\sum_{s,t=-\infty }^{\infty }\left(\frac{(\rho_{0}-\rho_{I})\omega^{2}}{\mu_{0}}\int_{V_{st}}w(\text{y})G(\text{y}-\text{x})\;d\text{y}-\frac{\left(\mu_{0}-\mu_{I}\right)}{\mu_{0}}\int_{V_{st}}\nabla_{y}w(\text{y})\cdot \nabla_{y}G(\text{y}-\text{x})\;d\text{y}\right),$$

where ∇_*y*_=(∂/∂*y*_1_,∂/∂*y*_2_). Non-dimensionalizing using the scalings $\hat{\text{x}}=q\text{x},\hat{\text{y}}=q\text{y}$ and $\hat{w}=w/\hat{W}$, with $\hat{W}$ being a typical displacement magnitude, and noting that Green’s function is already non-dimensional, the lattice vector in scaled coordinates becomes ***p***=*s****l***_1_+*t****l***_2_ and the cross-sectional area of the periodic cell is $R=A_{1}B_{2}$. At this point, it appears convenient to define the volume fraction per unit span in the *x*_3_-direction, ϕ=|D|/R, where |*D*| is the (non-dimensional) cross-sectional area of the fibre.

Upon dropping the hat notation, the non-dimensional integral equation takes the form

2.5 $$w(\text{x})=\sum_{s,t=-\infty }^{\infty }\left((1-d)\varepsilon^{2}\int_{V_{st}}w(\text{y})G_{\varepsilon }(\text{y}-\text{x})\;d\text{y}-(1-m)\int_{V_{st}}\nabla_{y}w(\text{y})\cdot \nabla_{y}G_{\varepsilon }(\text{y}-\text{x})\;d\text{y}\right),$$

where *d*=*ρ*_*I*_/*ρ*_0_ and *m*=*μ*_*I*_/*μ*_0_ are the contrasts in mass density and shear moduli, *ε*=*qk*_0_ and *G*_*ε*_(***x***−***y***)=(1/4*i*)*H*_0_(*ε*|***x***−***y***|). Note that as ε→0,

2.6 $$G_{\varepsilon }(\text{x}-\text{y})∼\frac{1}{2\pi }\ln(|\text{x}-\text{y}|)+\gamma_{c}+O(\varepsilon )=G_{0}(\text{x}-\text{y})+\gamma_{c}+O(\varepsilon ),$$

where $\gamma_{c}=(1/4\pi )(2\gamma_{e}-i\pi -2\log2)$ and *γ*_*e*_≃0.577216… is Euler’s constant. Having already dropped hats, referring to (2.2), in non-dimensional coordinates the boundary of the fibre is, therefore, described by *r*=ℓ*f*(*θ*), *θ*∈[0,2*π*], where ℓ=*a*/*q*. The non-dimensional fibre cross section is easily shown to be

$$|D|=\int_{0}^{2\pi }\int_{0}^{\ell f(\theta )}r\;dr\;d\theta =\frac{\ell^{2}}{2}\int_{0}^{2\pi }f^{2}(\theta )\;d\theta$$

and the *volume fraction* of the fibre cross section within the periodic cell is then

2.7 $$\phi =\frac{|D|}{R}=\frac{\ell^{2}}{2R}\int_{0}^{2\pi }f^{2}(\theta )\;d\theta .$$

Therefore,

2.8 $$\ell =\sqrt{\frac{2R\phi }{\int_{0}^{2\pi }f^{2}(\theta )\;d\theta }}=\tau \sqrt{\phi }.$$

Although expansions are sought with respect to *ϕ* below, in some instances it is more convenient to work with ℓ initially. However, in the end using (2.8), general expressions are determined in terms of *ϕ*. Attention here is also restricted to the scenario where *τ* remains *O*(1), with respect to *ϕ*. Therefore, from (2.8),

2.9 $$\ell =O\left(\sqrt{\phi }\right).$$

In the homogenization regime, *ak*_0_≪1 and *ε*=*qk*_0_≪1 and it can be assumed here that *ak*_0_=*O*(*ε*). In [21,22], the regime where *ak*_0_≪1 but *ε* is not restricted to being small was considered. Note that this requires *ϕ*≪1. If one wished, the method introduced in the next section could be modified in order to consider this regime.

It is important to note that as they stand the infinite sums in (2.5) are not absolutely convergent. This is an issue that arises frequently in effective media problems where the material in question is of infinite extent. Sensible results can be derived by a number of different approaches. In PA, this issue was dealt with by allowing a small amount of ‘loss’ in the system. This means taking wavenumbers of the form *k*+*iη* where *η*≪1. Non-zero *η* ensures convergence (in this case of integrals that arise when carrying out the sum to integral step, as developed in PA) and the limit η→0 is then taken. The reader is referred to PA for further details.

## 3. The integral equation method

In PA, it was shown that setting *m*=1 leads to the result ρ_*_=(1−*ϕ*)+*dϕ* for the non-dimensional effective density (scaled on *ρ*_0_) in the quasi-static limit. This is an exact result in the separation of scales regime. Here, without loss of generality and in order to determine the effective shear modulus, set *d*=1 in (2.5). Differentiate both sides of the resulting integral equation with respect to *x*_*k*_,*k*=1,2 to yield

3.1 $$\frac{\partial w}{\partial x_{k}}(\text{x})=-(1-m)\sum_{s,t=-\infty }^{\infty }\left(\frac{\partial }{\partial x_{k}}\int_{V_{st}}\nabla_{y}w(\text{y})\cdot \nabla_{y}G_{\varepsilon }(\text{y}-\text{x})\;d\text{y}\right).$$

Now take ***x***∈*V*_*ab*_, i.e. within the (*a*, *b*)th fibre, which has position vector ***r***=*a****l***_1_+*b****l***_2_. By taking the Taylor expansion of Green’s function about the point ***y***=***p***=(*p*_1_, *p*_2_)=*s****l***_1_+*t****l***_2_ (i.e. about the centre of the (*s*, *t*)th fibre), (3.1) becomes

3.2 $$\begin{array}{rl} \partial_{x_{k}}w(\text{x}) & \;=(1-m)\sum_{\begin{array}{c} s,t=-\infty \\ \left(s,t)\neq (a,b\right) \end{array}}^{\infty }\left(\sum_{n=1}^{2}\sum_{i,j=0}^{\infty }W_{ij}^{\left(n\right)}(\text{p})(\partial_{y_{n}}\partial_{y_{k}}\partial_{y_{1}}^{i}\partial_{y_{2}}^{j}G_{\varepsilon }(\text{y}-\text{x})){|}_{\text{y}=\text{p}}\right)\\ & \;\;-(1-m)\partial_{x_{k}}\int_{V_{ab}}\nabla_{y}w(\text{y})\cdot \nabla_{y}G_{\varepsilon }(\text{y}-\text{x})d\text{y}, \end{array}$$

where $\partial_{y_{k}}^{i}$ denotes the *i*th derivative with respect to *y*_*k*_ and

3.3 $$W_{ij}^{\left(k\right)}(\text{p})=\int_{V_{st}}\frac{1}{i!j!}{\left(y_{1}-p_{1}\right)}^{i}{\left(y_{2}-p_{2}\right)}^{j}\frac{\partial w}{\partial y_{k}}(\text{y})\;d\text{y}=O(\phi^{(i+j+2)/2}).$$

The variables introduced as $W_{ij}^{\left(k\right)}(\text{p})$ can be thought of as *displacement-gradient moments* of order *i*+*j*, recalling that ϕ=|D|/R. The order of these moments has been deduced using the fact that, since *w* is a piecewise smooth function, *w* and all its derivatives will be *O*(1).

Note that the term for (*s*,*t*)=(*a*,*b*) is not included in the summation, nor has the Taylor series of Green’s function been taken in this term. This is because Green’s function is singular in the domain *V*_*ab*_ since ***x*** is contained in this region. The assumption that one can Taylor expand Green’s function puts restrictions upon the parameters *ε* and *ϕ*. Either (i) *ε*≪1 in which case *ϕ* is unrestricted *or* (ii) *ε*=*O*(1) and then *ϕ*≪1 is required. Here, only scenario (i) is considered; see also the discussion at the end of the last section.

Proceed now by defining the operation $L_{\delta \xi }[(*)]$ as the act of multiplying each side of equation (*) by (*x*_1_−*r*_1_)^*δ*^(*x*_2_−*r*_2_)^*ξ*^/(*δ*!*ξ*!) and integrating in the ***x*** plane over the domain *V*_*ab*_, where ***r***=(*r*_1_,*r*_2_). Hence, consider $L_{\delta \xi }$[(3.2)] and subsequently Taylor expand Green’s function and its derivatives about ***x***=***r*** to obtain, after some rearrangement

3.4 $$\begin{array}{rl} & \frac{W_{\delta \xi }^{\left(k\right)}(\text{r})}{\left(m-1\right)}+\sum_{\text{p}\neq \text{r}}\left(\sum_{n=1}^{2}\sum_{i,j,\alpha ,\beta =0}^{\infty }W_{ij}^{\left(n\right)}(\text{p})C_{\delta \xi \alpha \beta }{\left(\partial_{y_{n}}\partial_{y_{k}}\partial_{y_{1}}^{i+\alpha }\partial_{y_{2}}^{j+\beta }G_{\varepsilon }(\text{y}-\text{x}))\right|}_{\text{x}=\text{r},\text{y}=\text{p}}\right)=A_{\delta \xi }^{\left(k\right)}(\text{r}), \end{array}$$

where the property ∂*G*_*ε*_/∂*x*_*k*_=−∂*G*_*ε*_/∂*y*_*k*_ has been employed. Here, the outermost summation notation refers to summing over every fibre location ***p***=(*p*_1_,*p*_2_) except for ***p***=***r***. Furthermore, the following terms have been defined:

3.5 $$\begin{array}{rl} A_{\delta \xi }^{\left(k\right)}(\text{r}) & \;=\int_{V_{ab}}\frac{{\left(x_{1}-r_{1}\right)}^{\delta }{\left(x_{2}-r_{2}\right)}^{\xi }}{\delta !\xi !}\partial_{x_{k}}\int_{V_{ab}}\nabla_{y}w(\text{y})\cdot \nabla_{y}G_{\varepsilon }(\text{y}-\text{x})\;d\text{y}\;d\text{x}\\ & \;=O(\phi^{(\delta +\xi +2)/2}) \end{array}$$

and

3.6 $$\begin{array}{rl} C_{\delta \xi \alpha \beta } & \;=\int_{V_{ab}}\frac{{\left(-1\right)}^{\alpha +\beta }}{\alpha !\beta !\delta !\xi !}{\left(x_{1}-r_{1}\right)}^{\delta +\alpha }{\left(x_{2}-r_{2}\right)}^{\xi +\beta }\;d\text{x} \end{array}$$

3.7 $$\begin{array}{rl} & \;=\int_{0}^{2\pi }\int_{0}^{\ell f(\hat{\theta })}\frac{{\left(-1\right)}^{\alpha +\beta }R^{\delta +\alpha +\xi +\beta +1}}{\alpha !\beta !\delta !\xi !}{\left(\cos\Theta \right)}^{\delta +\alpha }{\left(\sin\Theta \right)}^{\xi +\beta }\;dR\;d\Theta \\ & \;=\frac{{\left(-1\right)}^{\alpha +\beta }\ell^{\delta +\alpha +\xi +\beta +2}}{\alpha !\beta !\delta !\xi !(\delta +\alpha +\xi +\beta +2)}\int_{0}^{2\pi }{\left[f(\Theta )\right]}^{\delta +\alpha +\xi +\beta +2}{\left(\cos\Theta \right)}^{\delta +\alpha }{\left(\sin\Theta \right)}^{\xi +\beta }\;d\Theta \\ & \;=(\ell^{\delta +\alpha +\xi +\beta +2}){\hat{C}}_{\delta \xi \alpha \beta }, \end{array}$$

where the local polar coordinate system $x_{1}-r_{1}=R\cos\Theta ,x_{2}-r_{2}=R\sin\Theta$ has been defined and where (2.8) is used in order to define ${\hat{C}}_{\delta \xi \alpha \beta }=O(1)$. As was shown in PA, the influence of the cross-sectional shape of the fibre is embedded *solely* in terms of the constants ${\hat{C}}_{\delta \xi \alpha \beta }$ and the shape factors $A_{\delta \xi }^{\left(k\right)}$, the form of which shall be considered shortly.

### (a) The shape factor

The term $A_{\delta \xi }^{\left(k\right)}$ incorporating the fibre cross section in (3.5) appears to possess a singularity at ***y***=***x*** due to the presence of derivatives of Green’s function in the integrand. However, as was shown in PA, this apparent singular contribution is found to be zero by splitting the domain *V*_*ab*_ up into a non-singular part *V*_*ab*_∖*C*_*ψ*_ and apparently singular part *C*_*ψ*_, where *C*_*ψ*_ is a disc of radius *ψ*≪1 with origin ***y***=***x***. Therefore, all that remains to consider are integrals of the type

3.8 $$A_{\delta \xi }^{\left(k\right)}(\text{r})=\lim_{\psi \to 0}\int_{V_{ab}}\frac{{\left(x_{1}-r_{1}\right)}^{\delta }{\left(x_{2}-r_{2}\right)}^{\xi }}{\delta !\xi !}\partial_{x_{k}}\int_{V_{ab}\setminus C_{\psi }}\nabla_{y}w(\text{y})\cdot \nabla_{y}G_{\varepsilon }(\text{y}-\text{x})\;d\text{y}\;d\text{x}.$$

Once again employing the property ∂*G*_*ε*_/∂*x*_*k*_=−∂*G*_*ε*_/∂*y*_*k*_, the *x*_*k*_ derivative may be taken inside the ***y*** integral, and as the range of integration does not include the region where *G*(***y***−***x***) is singular, exchanging the order of integration is permissable. Retaining the leading-order Green’s function as ε→0,

3.9 $$A_{\delta \xi }^{\left(k\right)}(\text{r})=-\lim_{\psi \to 0}\int_{V_{ab}\setminus C_{\psi }}\partial_{y_{1}}w(\text{y})\partial_{y_{k}}\partial_{y_{1}}J_{\delta \xi }(\text{y})+\partial_{y_{2}}w(\text{y})\partial_{y_{k}}\partial_{y_{2}}J_{\delta \xi }(\text{y})\;d\text{y}+O(\varepsilon )$$

where

3.10 $$J_{\delta \xi }(\text{y})=\int_{V_{ab}}\frac{{\left(x_{1}-r_{1}\right)}^{\delta }{\left(x_{2}-r_{2}\right)}^{\xi }}{\delta !\xi !}G_{0}(\text{y}-\text{x})\;d\text{x},$$

recalling that *G*_0_ was defined in (2.6).

It is convenient here to represent (3.10) as a series expansion, i.e.

3.11 $$J_{\delta \xi }=D_{00}^{\delta \xi }+D_{10}^{\delta \xi }(y_{1}-r_{1})+D_{01}^{\delta \xi }(y_{2}-r_{2})+\sum_{p=2}^{P+2}\sum_{q=0}^{p}\left(\frac{D_{(p-q)q}^{\delta \xi }}{(p-q)!q!}{\left(y_{1}-r_{1}\right)}^{p-q}{\left(y_{2}-r_{2}\right)}^{q}\right)$$

so that displacement gradient moments naturally arise in (3.9). A procedure for obtaining the coefficients $D_{pq}^{\delta \xi }$ for a given fibre cross section is outlined in appendix A. The order of truncation, P+2 is governed by the shape function *f* involved. For elliptical fibres P=δ+ξ is an exact result for example. For arbitrarily shaped cross sections P→∞ generally. It is noted that second derivatives of (3.10) make up the components of the generalized Hill tensor [23,24]. Its representation via a polynomial expansion as in (3.11) will be discussed in a forthcoming article by the authors.

Substituting (3.11) into (3.9) and adjusting the indices one obtains

3.12 $$A_{\delta \xi }^{\left(1\right)}(\text{r})=-\sum_{p=0}^{P}\sum_{q=0}^{p}\left(D_{(p-q+2)q}^{\delta \xi }W_{(p-q)q}^{\left(1\right)}(\text{r})+D_{\left(p-q+1)(q+1\right)}^{\delta \xi }W_{(p-q)q}^{\left(2\right)}(\text{r})\right)+O(\varepsilon )$$

and

3.13 $$A_{\delta \xi }^{\left(2\right)}(\text{r})=-\sum_{p=0}^{P}\sum_{q=0}^{p}\left(D_{\left(p-q+1)(q+1\right)}^{\delta \xi }W_{(p-q)q}^{\left(1\right)}(\text{r})+D_{\left(p-q)(q+2\right)}^{\delta \xi }W_{(p-q)q}^{\left(2\right)}(\text{r})\right)+O(\varepsilon ).$$

Note that $D_{00}^{\delta \xi }$, $D_{10}^{\delta \xi }$ and $D_{01}^{\delta \xi }$ do not arise in the shape factor as they do not survive second-order differentiation. In the case of fibres with circular cross sections, *f*(*θ*)=1 and it follows that ${\hat{C}}_{\delta \xi \alpha \beta }=0$ if *δ*+*ξ*+*α*+*β* is odd. As $A_{\delta \xi }^{\left(k\right)}=O(\phi^{(\delta +\xi +2)/2})=W_{\delta \xi }^{\left(k\right)}$, equations (3.12)–(3.13) lead to the conclusion that $D_{mn}^{\delta \xi }=O(\phi^{(\delta +\xi -m-n+2)/2})$.

Equations (3.12) and (3.13) coupled with (3.4) give rise to an infinite homogeneous system of linear equations for the displacement gradient moments $W_{\delta \xi }^{\left(k\right)}$. A wave-like ansatz for these moments is now posed.

### (b) Wave-like solutions

Noting (2.9), plane wave type solutions of (3.4) are sought of the form

3.14 $$W_{ij}^{\left(k\right)}(\text{r})={\hat{W}}_{ij}^{k}\ell^{i+j+2}\exp(i\Gamma \cdot \text{r}),$$

with Γ(Θ)=εγ(Θ)(cos⁡Θ,sin⁡Θ) being the non-dimensional effective wavenumber (scaled on *q*) in the direction of the angle subtended from the *x*_1_-axis, *Θ*. Furthermore, define

3.15 $$\begin{array}{r} D_{mn}^{\delta \xi }=\ell^{\delta +\xi -m-n+2}{\hat{D}}_{mn}^{\delta \xi }. \end{array}$$

Therefore, using (2.8), (3.7), (3.12)–(3.15) in (3.4) (recalling *k*=1,2) and recalling (2.9), at leading order (with respect to *ε*)

3.16 $$\begin{array}{rl} & \frac{{\hat{W}}_{\delta \xi }^{\left(1\right)}}{\left(1-m\right)}-\sum_{i,j,\alpha ,\beta =0}^{\infty }\left({\hat{C}}_{\delta \xi \alpha \beta }{\left(\tau \phi^{1/2}\right)}^{i+j+\alpha +\beta +2}\{{\hat{W}}_{ij}^{\left(1\right)}A[i+\alpha +2,j+\beta \right]\\ & \;+{\hat{W}}_{ij}^{\left(2\right)}A[i+\alpha +1,j+\beta +1]\})=\sum_{p=0}^{P}\sum_{q=0}^{p+2}\left({\hat{D}}_{(p-q+2)q}^{\delta \xi }{\hat{W}}_{(p-q)q}^{\left(1\right)}+{\hat{D}}_{\left(p-q+1)(q+1\right)}^{\delta \xi }{\hat{W}}_{(p-q)q}^{\left(2\right)}\right) \end{array}$$

and

3.17 $$\begin{array}{rl} & \frac{{\hat{W}}_{\delta \xi }^{\left(2\right)}}{\left(1-m\right)}-\sum_{i,j,\alpha ,\beta =0}^{\infty }\left({\hat{C}}_{\delta \xi \alpha \beta }{\left(\tau \phi^{1/2}\right)}^{i+j+\alpha +\beta +2}\{{\hat{W}}_{ij}^{\left(1\right)}A[i+\alpha +1,j+\beta +1\right]\\ & \;+{\hat{W}}_{ij}^{\left(2\right)}A[i+\alpha ,j+\beta +2]\})=\sum_{p=0}^{P}\sum_{q=0}^{p+2}\left({\hat{D}}_{\left(p-q+1)(q+1\right)}^{\delta \xi }{\hat{W}}_{(p-q)q}^{\left(1\right)}+{\hat{D}}_{\left(p-q)(q+2\right)}^{\delta \xi }{\hat{W}}_{(p-q)q}^{\left(2\right)}\right), \end{array}$$

where

3.18 $$A[m,n]=\lim_{\varepsilon \to 0}\sum_{\text{u}\neq \text{0}}\partial_{u_{1}}^{m}\partial_{u_{2}}^{n}G_{\varepsilon }(\text{u})\exp(i\Gamma \cdot \text{u})$$

is a lattice sum, which is discussed in the next section.

### (c) Lattice sums

First pick out the singular, non-integrable behaviour of the derivatives of Green’s function in the lattice sum (3.18), writing

3.19 $$\partial_{u_{1}}^{m}\partial_{u_{2}}^{n}G_{\varepsilon }(\text{u})∼S[m,n]+O(1)\;\mathrm{as}|\text{u}|\to 0,$$

identifying *S*[*m*,*n*] as the singular part of the derivative. Then for a given *m*,*n*, (3.18) can be written as

3.20 $$A[m,n]=\lim_{\varepsilon \to 0}\sum_{\text{u}\neq \text{0}}[(\partial_{u_{1}}^{m}\partial_{u_{2}}^{n}G_{\varepsilon }(\text{u})-S[m,n])\exp(i\Gamma \cdot \text{u})]+L_{0}[m,n],$$

with $L_{0}[m,n]=\lim_{\varepsilon \to 0}L[m,n]$ where

3.21 $$L[m,n]=\sum_{\text{u}\neq \text{0}}S[m,n]\exp(i\Gamma \cdot \text{u}).$$

Therefore,

3.22 $$L_{0}[m,n]=\sum_{\text{u}\neq \text{0}}S_{0}[m,n],$$

with $\partial_{u_{1}}^{m}\partial_{u_{2}}^{n}G_{0}(\text{u})∼S_{0}[m,n]+O(1)$ as |u|→0. The first term of (3.20) can be turned into an integral in the same manner as in Sec. 3(a) of PA. It transpires that when this step is applied with *m*=*i*+*α*+*p* and *n*=*j*+*β*+*q*, such that (*p*,*q*)∈{(2,0),(1,1),(0,2)}, one is left with an integral that is *O*(1) with respect to *ε*, multiplied by a factor *ε*^*i*+*j*+*α*+*β*^. Therefore, the only term from this step that remains in the limit ε→0 is when *i*=*j*=*α*=*β*=0. Thus,

3.23 $$A[i+\alpha +p,j+\beta +q]=\left\{ \begin{array}{ll} I_{0}[p,q]+L_{0}[p,q] & \text{if }i=j=\alpha =\beta =0\\ L_{0}[i+\alpha +p,j+\beta +q] & \text{otherwise,} \end{array}\right.$$

where

3.24 $$I_{0}[p,q]=\lim_{\varepsilon \to 0}\frac{1}{R}\int_{-\infty }^{\infty }\int_{-\infty }^{\infty }\left(\partial_{u_{1}}^{p}\partial_{u_{2}}^{q}G_{\varepsilon }(\text{u})-S[m,n]\right)\exp(i\Gamma \cdot \text{u})\;d\text{u},$$

recalling that (*p*,*q*)∈{(2,0),(1,1),(0,2)}.

Recalling the definition of ***Γ*** in and below (3.14), taking *Θ*=0 and defining *γ*(0)=*γ*_1_, so that the wavevector is ***Γ***=*ε*(*γ*_1_,0) with *γ*_1_ being the effective wavenumber in the *x*_1_-direction, it was shown in the appendices to PA that (using the present notation)

3.25 $$I_{0}[2,0]=\frac{1}{\gamma_{1}^{2}-1}=g(\gamma_{1})\;\mathrm{and}\;I_{0}[1,1]=I_{0}[0,2]=0.$$

One can show that the integral in (3.24) can be evaluated via stationary phase. Alternatively, as was discussed at the end of §2 one can add loss to the system. Further details of this approach can be found in the appendix of PA.

If one wishes to determine the effective wavenumber in the *x*_2_-direction (seeking $\mu_{2}^{*}$ instead of $\mu_{1}^{*}$), it is straightforward to rotate the material by *π*/2. Performing this action leaves the above integrals unchanged, the wave is considered to propagate in the (new) *x*_1_-direction, merely using notation *γ*_2_ instead of *γ*_1_ to indicate the wavenumber associated with the new material direction of propagation.

One can determine a number of results for specific *L*_0_[*m*,*n*] straightforwardly. Firstly, since the singular part of *G*_0_ satisfies Laplace’s equation (except at the singular point, which is not important in the lattice summations as they exclude this point), the lattice sums will satisfy

3.26 $$L_{0}[m+2,n]=-L_{0}[m,n+2]\;\forall \;m,n\in N.$$

Furthermore, only cases where *both* *m* *and* *n* are even will give a non-zero *L*_0_[*m*,*n*]. For example, consider the case of *L*_0_[1,2]=−*L*_0_[3,0] due to (3.26). The associated *S*_0_[*m*,*n*] takes the form

$$\begin{array}{rl} S_{0}[1,2]=-S_{0}[3,0] & \;=-\frac{\partial^{3}}{\partial u_{1}^{3}}\left(\frac{1}{2\pi }\ln|\text{u}|\right)\\ & \;=\frac{3u_{1}u_{2}^{2}-u_{1}^{3}}{\pi |\text{u}{|}^{3}}. \end{array}$$

When this is employed in the lattice sum (3.22), the odd powers of *u*_1_ in the numerator ensure that the summation is zero. This same reasoning gives *L*_0_[*m*,*n*]=0 whenever *m*+*n* is odd. It is shown in appendix B how *L*_0_[*m*,*n*] can be straightforwardly determined when it is non-zero, i.e. when *m*+*n* is even.

### (d) The asymptotic system in *ϕ*

Using (3.23) in (3.16) and (3.17) the leading-order system, with respect to *ε*, is

3.27 $$\begin{array}{rl} & \frac{{\hat{W}}_{\delta \xi }^{\left(1\right)}}{\left(1-m\right)}-{\hat{C}}_{\delta \xi 00}\tau^{2}\phi {\hat{W}}_{00}^{\left(1\right)}g(\gamma_{1})\\ & \;\;-(1-m)\sum_{i,j,\alpha ,\beta =0}^{\infty }\left({\hat{C}}_{\delta \xi \alpha \beta }{\left(\tau \phi^{1/2}\right)}^{i+j+\alpha +\beta +2}\{{\hat{W}}_{ij}^{\left(1\right)}L_{0}[i+\alpha +2,j+\beta \right]\\ & \;+{\hat{W}}_{ij}^{\left(2\right)}L_{0}[i+\alpha +1,j+\beta +1]\})=\sum_{p=0}^{P}\sum_{q=0}^{p+2}\left({\hat{D}}_{(p-q+2)q}^{\delta \xi }{\hat{W}}_{(p-q)q}^{\left(1\right)}+{\hat{D}}_{\left(p-q+1)(q+1\right)}^{\delta \xi }{\hat{W}}_{(p-q)q}^{\left(2\right)}\right) \end{array}$$

and

3.28 $$\begin{array}{rl} & \frac{{\hat{W}}_{\delta \xi }^{\left(2\right)}}{\left(1-m\right)}-\sum_{i,j,\alpha ,\beta =0}^{\infty }\left({\hat{C}}_{\delta \xi \alpha \beta }{\left(\tau \phi^{1/2}\right)}^{i+j+\alpha +\beta +2}\{{\hat{W}}_{ij}^{\left(1\right)}L_{0}[i+\alpha +1,j+\beta +1\right]\\ & \;+{\hat{W}}_{ij}^{\left(2\right)}L_{0}[i+\alpha ,j+\beta +2]\})=\sum_{p=0}^{P}\sum_{q=0}^{p+2}\left({\hat{D}}_{\left(p-q+1)(q+1\right)}^{\delta \xi }{\hat{W}}_{(p-q)q}^{\left(1\right)}+{\hat{D}}_{\left(p-q)(q+2\right)}^{\delta \xi }{\hat{W}}_{(p-q)q}^{\left(2\right)}\right). \end{array}$$

This is an eigenvalue problem with eigenvalues *γ*_1_ and associated eigenvectors comprising the moments ${\hat{W}}_{\delta \xi }^{\left(k\right)}$, noting that *γ*_1_ appears only in the term *g*(*γ*_1_). To make progress take expansions in the volume fraction, posing

3.29 $$\begin{array}{rl} {\hat{W}}_{\delta \xi }^{\left(1\right)} & \;=u_{\delta \xi }^{0}+u_{\delta \xi }^{1}\phi +u_{\delta \xi }^{2}\phi^{2}+\cdots , \end{array}$$

3.30 $$\begin{array}{rl} {\hat{W}}_{\delta \xi }^{\left(2\right)} & \;=v_{\delta \xi }^{0}+v_{\delta \xi }^{1}\phi +v_{\delta \xi }^{2}\phi^{2}+\cdots \end{array}$$

3.31 $$\begin{array}{rl} \mathrm{and}\;g(\gamma_{1}) & \;=\frac{h_{-1}}{\phi }+h_{0}+h_{1}\phi +h_{2}\phi^{2}+\cdots , \end{array}$$

where the form for *g* is motivated by (3.25) and the fact that $\gamma_{1}\to 1$ as ϕ→0.

Note the scaling in *ϕ* (or ℓ) of the wave-type solutions (3.14), so that moments whose indices total an odd number will have a fractional power at leading order in *ϕ*. For instance, $W_{10}^{\left(k\right)}(\text{r})=O(\phi^{3/2})$. This would suggest that, in general, powers of *ϕ*^1/2^ should be included in (3.29)–(3.31). However, restricting attention to shapes featuring a rotational symmetry of *π*, henceforth referred to as *centrally symmetric* shapes, and by observing the properties of the integrals in appendix A it can be shown that such fractional powers are not required in the expansions. Proof of this is given in appendix C, and the remainder of the methodology shall be discussed in the context of the restriction of fibre cross sections to central symmetry.

Given the scalings involved, using (3.25) and $\gamma_{1}^{2}=1/\mu_{1}^{*}$, (3.31) gives

3.32 $$\mu_{1}^{*}=\frac{h_{-1}+h_{0}\phi +h_{1}\phi^{2}+h_{2}\phi^{3}+\cdots }{h_{-1}+(1+h_{0})\phi +h_{1}\phi^{2}+h_{2}\phi^{3}+\cdots }.$$

Note the form of this expression, i.e. every coefficient at each order in the polynomials in the numerator and denominator is the same except for the *O*(*ϕ*) term. The concern of the next section is the determination of the coefficients *h*_*j*_ from the linear system.

## 4. Determining explicit forms for the effective properties

Equation (3.32) provides an explicit form for the effective shear modulus, as a rational function in *ϕ*, providing the coefficients *h*_*j*_ can be determined. Note that the approximation provided in PA was exactly this with both numerator and denominator truncated at *O*(*ϕ*). Here, the objective is to determine higher-order coefficients for a variety of fibre cross sections.

There is a straightforward algorithmic mechanism for determining the coefficients *h*_*j*_. Hereon in the term ‘(*δξ*) equations’ shall refer to (3.27) and (3.28) for fixed *δ* and *ξ*. Start by noting that expressions for the *h*_*j*_ coefficients arise by considering the first (00) equation, (3.27), using expansions (3.29)–(3.31) and equating each order in *ϕ* of the resulting equation. To determine all coefficients up to *h*_*N*_, say, the first (00) equation must be considered up to order *ϕ*^*N*+1^. Each order provides an expression for a coefficient *h*_*j*_ in terms of the eigenvector components $u_{00}^{j}$, lattice sums, coefficients of the shape factors and possibly components from other moments $u_{\delta \xi }^{j}$ and $v_{\delta \xi }^{j}$. By tracking which terms from the displacement gradient moments appear in this highest-order equation, one can then see which truncation with respect to the choices of indices (*δξ*) needs to be made and hence which additional (*δξ*) equations need to be considered in order to solve the linear system for the required terms $u_{\delta \xi }^{j}$ and $v_{\delta \xi }^{j}$ and hence subsequently *h*_*j*_. The following examples illustrate the implementation of this scheme; orderings now refer to *ϕ*.

### (a) Elliptical cylindrical fibres

Consider elliptical cylindrical fibres with semi-axes *a* and *b* in the *x*_1_ and *x*_2_ directions, respectively. At *O*(1), noting that ${\hat{C}}_{0000}\tau^{2}=1$, where we recall that *τ* is defined for a given shape in (2.8) and writing without loss of generality that $u_{00}^{0}=1$, it is determined that

4.1 $$h_{-1}=\frac{a+mb}{\left(1-m)(a+b\right)}.$$

It transpires that the second (00) equation yields $v_{00}^{0}=0$. The *O*(*ϕ*) terms give (upon using the properties of the lattice sum)

$$\frac{u_{00}^{1}}{\left(1-m\right)}=h_{-1}u_{00}^{1}+h_{0}u_{00}^{0}+u_{00}^{0}L[2,0]+\frac{bu_{00}^{1}}{a+b}\;,$$

and after using (4.1) this gives

4.2 $$0=(L[2,0]+h_{0})u_{00}^{0}\;\;⟹\;\;h_{0}=-L[2,0],$$

while the second (00) equation to *O*(*ϕ*) gives $v_{00}^{1}=0$. Continuing in this vein, (3.32) takes the form

4.3 $$\mu_{j}^{*}=\frac{p_{0j}+(1/2)(S_{j}-1)\phi -p_{2j}C_{4j}\phi^{2}-p_{3j}C_{6j}\phi^{3}-p_{4j}C_{4j}^{2}\phi^{4}+\cdots }{p_{0j}+(1/2)(S_{j}+1)\phi -p_{2j}C_{4j}\phi^{2}-p_{3j}C_{6j}\phi^{3}-p_{4j}C_{4j}^{2}\phi^{4}+\cdots },$$

where *L*_0_[2,0]=−*L*_0_[0,2]=−(*S*_*j*_−1)/2, *C*_4*j*_ and *C*_6*j*_ depend upon the fourth- and sixth-order lattice sums, respectively, and the *p*_*ij*_ coefficients are rational functions in *m* and also depend on aspect ratio. Their forms are too lengthy to be given here but can straightforwardly be derived by implementing the algorithm above. As one should expect through checking for consistency with other leading-order approximations

4.4 $$p_{01}=h_{-1}=\frac{a+mb}{\left(1-m)(a+b\right)}\;\mathrm{and}\;p_{02}=\frac{b+ma}{\left(1-m)(a+b\right)}.$$

For the specific case of fibres arranged on a square lattice, one finds that

4.5 $$\mu_{j}^{*}=\frac{p_{0j}-(1/2)\phi -p_{2j}C_{4j}\phi^{2}-p_{4j}C_{4j}^{2}\phi^{4}+\cdots }{p_{0j}+(1/2)\phi -p_{2j}C_{4j}\phi^{2}-p_{4j}C_{4j}^{2}\phi^{4}+\cdots }.$$

Details of how higher-order terms are derived will be given explicitly in the next section for the case of circular cross sections.

### (b) Circular cylindrical fibres

To illustrate specific details of the derivation of higher-order terms, take the circular cross-sectional case, *a*=*b*=1 for which the details of the last section are clearly valid. Consider the first (00) equation to *O*(*ϕ*^2^). After evaluating the ${\hat{C}}_{00\alpha \beta }$ coefficients in the quadruple sum, using the relations (3.26) and the rule involving odd indices within the lattice sum, in addition to the result from the previous section that $v_{00}^{1}=0$ and the forms (4.1) and (4.2) for *h*_−1_ and *h*_0_, the equation reduces to

4.6 $$0=h_{1}u_{00}^{0}+\frac{1}{\pi }(u_{20}^{0}-u_{02}^{0}-v_{11}^{0})L[4,0].$$

This illustrates how higher-order moment terms arise, in this case $u_{20}^{0}$, $u_{02}^{0}$ and $v_{11}^{0}$, and therefore motivates which equations need to be studied subsequently. In this case, it means that the (11), (20) and (02) equations must be considered at leading order with respect to *ϕ* in order to obtain *h*_1_. Using the naming convention that the collective set of (*ij*) equations where *i*+*j*=*n* shall be referred to as the order *n* equations, here the interest is therefore in the order 2 equations.

It transpires that these order 2 equations partition into two non-trivial decoupled sub-systems: the first (20) and (02) equations and the second (11) equation form a system in ${\hat{W}}_{20}^{\left(1\right)}$, ${\hat{W}}_{02}^{\left(1\right)}$ and ${\hat{W}}_{11}^{\left(2\right)}$, while the second (20) and (02) equations and the first (11) equation form a system in ${\hat{W}}_{20}^{\left(2\right)}$, ${\hat{W}}_{02}^{\left(2\right)}$ and ${\hat{W}}_{11}^{\left(1\right)}$. The latter subsystem is homogeneous with respect to the leading-order coefficients of the moments featured, while (4.6) illustrates how the former contributes to the process of obtaining *h*_1_. Hence, making use of (4.1) for *a*=*b*=1 and all other solutions from prior orders of *ϕ*, the leading-order equations of the former sub-system take the form

$$\begin{array}{rl} 0 & \;=\frac{1}{16}u_{00}^{0}\left(\frac{1+m}{1-m}-1\right)+\frac{1}{8}u_{02}^{1}+\left(\frac{7}{8}-\frac{1}{1-m}\right)u_{20}^{1}+\frac{1}{8}v_{11}^{1},\\ 0 & \;=\frac{1}{16}u_{00}^{0}\left(\frac{1+m}{1-m}+1\right)+\left(\frac{1}{8}-\frac{1}{1-m}\right)u_{02}^{1}-\frac{1}{8}u_{20}^{1}+\frac{1}{8}v_{11}^{1}\\ \mathrm{and}\;0 & \;=-\frac{1}{8}u_{00}^{0}+\frac{1}{2}u_{02}^{1}+\frac{1}{2}u_{20}^{1}+\left(\frac{1}{2}-\frac{1}{1-m}\right)v_{11}^{1}. \end{array}$$

Solving these equations gives

$$u_{20}^{1}=u_{02}^{1}=\frac{u_{00}^{0}}{8}\;\mathrm{and}\;v_{11}^{1}=0,$$

and hence (4.6) gives *h*_1_=0.

Proceeding to general orders, with the aid of a symbolic package such as Mathematica, for general parallelogram lattices, results for circular cylindrical fibres take the form

4.7 $$\mu_{j}^{*}=\frac{\begin{array}{c} 1+(S_{j}-1)M\phi -(M^{2}C_{4j}^{2}/3\pi^{2})\phi^{4}-(M^{2}C_{6j}^{2}/720\pi^{4})\phi^{6}-\\ (M^{3}C_{4j}^{2}C_{6j}/18\pi^{4})\phi^{7}-(M^{2}C_{8j}^{2}/\pi^{6})\phi^{8}+\cdots \end{array}}{\begin{array}{c} 1+(S_{j}+1)M\phi -(M^{2}C_{4j}^{2}/3\pi^{2})\phi^{4}-(M^{2}C_{6j}^{2}/720\pi^{4})\phi^{6}-\\ (M^{3}C_{4j}^{2}C_{6j}/18\pi^{4})\phi^{7}-(M^{2}C_{8j}^{2}/\pi^{6})\phi^{8}+\cdots \end{array}},$$

where M=(1−m)/(1+m). Note that the sixth-order lattice sum is zero for square lattices, hence *C*_6*j*_=0 which is why order *ϕ*^6^ and *ϕ*^7^ terms appear in the general case and not the square lattice case.

In the special case when circular cylindrical fibres on a square lattice, expressions for the effective shear moduli take the form

4.8 $$\mu_{j}^{*}=\frac{1-M\phi -(M^{2}C_{4j}^{2}/3\pi^{2})\phi^{4}-(M^{2}C_{8j}^{2}/\pi^{6})\phi^{8}+\cdots }{1+M\phi -(M^{2}C_{4j}^{2}/3\pi^{2})\phi^{4}-(M^{2}C_{8j}^{2}/\pi^{6})\phi^{8}+\cdots }.$$

This is consistent with the form of higher-order terms outlined in the conclusion of PA.

### (c) More general fibre cross sections

Although rather lengthy, analytical forms for the effective shear moduli associated with general cross sections *can* be obtained. The procedure above can be followed and certainly in a symbolic package such as Mathematica, forms can be derived. However, such expressions are too lengthy and cumbersome in general to be provided here. Importantly, for a given cross-sectional shape, using the above procedure, a rational function approximation for the effective properties as a function of *ϕ* can be derived and subsequently used to great utility. In the following section, we discuss results obtained for shapes more general than elliptical and validate the scheme with the classical method of asymptotic homogenization (MAH). The argument for using the present scheme over the MAH (or other methods) is that this integral equation methodology can yield explicit forms, particularly when some aspects of the medium are fixed (e.g. square lattice, fixed *m*), of the effective moduli, retaining dependence on *ϕ*. Such forms can then be used with great rapidity in models without the need for recourse to finite-element simulations as soon as one changes, for example, the volume fraction, as is required in the MAH for general shapes, for example.

## 5. Results

The implementation of the above methodology is now described for a range of geometries in the case of a shear contrast of *m*=18.75 (corresponding to the case of graphite fibres in epoxy, for example). To fix ideas and since general cross sections are of principal interest here, attention is restricted to the case of square lattices. Extension to other lattices is straightforward. Results derived using the methodology shall be compared with those obtained using the MAH [1]. It transpires that the effective antiplane shear moduli (when scaled on that of the host medium) as determined by the MAH can be written in the form (see eqns (3.25) and (3.26) of [1])

5.1 $$\mu_{1}^{*}=1+(m-1)(\phi +H_{11})\;\mathrm{and}\;\mu_{2}^{*}=1+(m-1)(\phi +H_{22}),$$

where the tensor components of **H** may be determined as

5.2 $$\text{H}=\int_{V_{ab}}\nabla_{\xi }\text{N}\;d\xi =\int_{\partial V_{ab}}\text{N}⊗\text{n}\;ds=\left[\begin{array}{cc} H_{11} & H_{12}\\ H_{21} & H_{22} \end{array}\right].$$

Here, ***n*** is the outer unit normal to the fibre boundary ∂*V*_*ab*_, ***ξ*** is the short lengthscale of the problem, and ***N***=(*N*_1_,*N*_2_) is the solution to the associated *cell problem*. In the case of orthotropy, *H*_12_=*H*_21_=0, and if the fibre cross section has a rotational symmetry of *π*/2, *H*_11_=*H*_22_ and thus $\mu_{1}^{*}=\mu_{2}^{*}$. Generally for fibres of non-circular cross section, the finite-element method (FEM) is employed to solve the cell problem. Indeed here, COMSOL multiphysics is used to solve the cell problem for antiplane shear. The methods are compared by plotting the components *H*_11_ and *H*_22_ of the **H**-tensor. To do this for the integral equation method (IEM), the shear moduli are determined using that scheme and then the components of the **H**-tensor are computed using expressions (5.1). It will be shown that these components provide a very sensitive measure of the accuracy of the IEM and generally speaking, the method provides excellent accuracy even at extremely high volume fractions, for relatively low-order approximations of the rational function form. Note that when results are presented, we will use the term *IEM order *n** to refer to the rational function form (3.32) when order *n* polynomials in *ϕ* are retained in the numerator and denominator. In particular, *IEM order 1* refers to the scheme employed in PA, i.e. (1.3).

### (a) Circular cylindrical fibres

**Figure 3.**
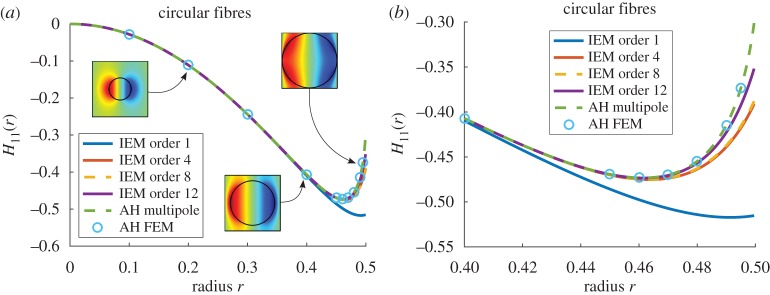
Plot of *H*_11_(*r*) for *m*=18.75 when circular fibres are arranged on a square lattice, with associated solutions of the cell problem from the MAH inset (*a*) and a magnified plot for large radii (*b*). (Online version in colour.)

Consider circular fibres of radius *r*. Figure 3 compares results for *H*_11_(*r*)=*H*_22_(*r*) as obtained from the IEM at various orders, to those obtained using the MAH. Results are determined as a function of the scaled radius *r*=ℓ, related to the volume fraction *ϕ* via equation (2.8) for *f*(*θ*)=1 and R=1. Results for the MAH when using the multipole method [1] and the FEM are also plotted to illustrate that the FEM remains consistent with the multipole method. Agreement between the MAH and the IEM estimates is, in general, excellent until the radius begins to approach 0.5 when fibres become very close. Figure 3*b* focuses on values of the radius close to this packing limit. The highest-order IEM employed here (order 12) begins to deviate from the MAH estimate around *r*=0.48, although this is the deviation when studying the components of **H**. Generally, the impact of this deviation on *effective properties* is weaker as will be seen shortly.

### (b) Elliptical cylindrical fibres

**Figure 4.**
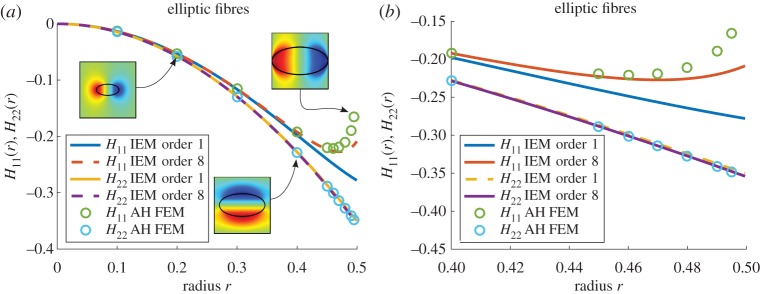
Plot of *H*_11_(*r*) and *H*_22_(*r*), where *r* is the major axis of the ellipse, for *m*=18.75 when elliptical fibres of aspect ratio 2 are arranged on a square lattice, with associated solutions of the cell problem from the MAH inset (*a*) and a magnified plot for large radii (*b*). (Online version in colour.)

Figure 4 illustrates results obtained in the case of elliptical fibres with aspect ratio (major axis divided by minor axis) of *ϵ*=2 and here *r* is the major axis. As figure 4*a* illustrates, agreement is excellent in general and it is only near the packing limit where the IEM and MAH results show any deviation, but only for *H*_11_ since this is the component that is affected by the near-neighbour interaction. Figure 4*b* magnifies this large radius region to clearly illustrate this effect. The order 8 IEM expression begins to show the upturn required in order to match the MAH at high radius for *H*_11_. The **H**-tensor components are a very sensitive measure of the accuracy of the IEM scheme however. It will be shown in the next section that when the effective properties are computed such high-order accuracy in these components is not required in order to provide good results.

### (c) Results for other fibre cross sections

**Figure 5.**
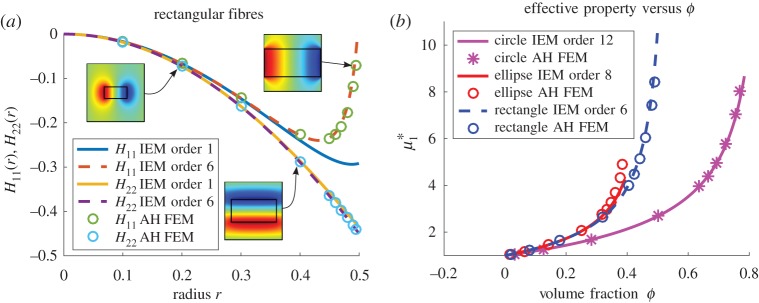
Plot of *H*_11_(*r*) and *H*_22_(*r*) for the situation when for *m*=18.75 and rectangular fibres of aspect ratio 2 are arranged on a square lattice, with associated solutions of the cell problem from the MAH inset (*a*) and a plot of the effective property $\mu_{1}^{*}$ versus volume fraction for circular, rectangular and elliptical fibre cross sections (*b*). (Online version in colour.)

**Figure 6.**
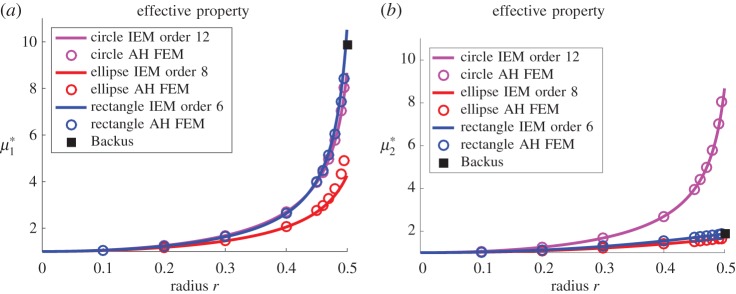
Plot of the effective antiplane shear moduli $\mu_{1}^{*}$ (*a*) and $\mu_{2}^{*}$ (*b*) versus fibre ‘radius’ for the situation when circular, elliptical and rectangular fibres are arranged on a square lattice, *m*=18.75. The black squares at *r*=1/2 indicate the layered media results as given in (5.3). (Online version in colour.)

The scheme is intended to work for fibres of general cross section. Having established promising results for fibres of elliptical cross section, it is now of interest to examine the efficacy of the method for more general cross sections and the polygonal case shall be considered first. In figure 5*a*, the results for *H*_11_(*r*) and *H*_22_(*r*) are examined in the case of rectangular-shaped fibres with an aspect ratio of 2 and where *r* is the long axis of the rectangle. Figure 5*b* illustrates results for the specific effective shear modulus $\mu_{1}^{*}$ for all three fibre shapes considered thus far (circular, elliptical and rectangular), when plotted against volume fraction, as opposed to fibre ‘radius’. Figure 6 plots both effective shear moduli $\mu_{1}^{*}$ and $\mu_{2}^{*}$ against fibre ‘radius’. As well as returning the results to the context of the actual physical-effective properties, these plots illustrate the extra sensitivity that the **H**-tensor components exhibit, i.e. in general even for quite small-order approximations the IEM appears to provide exceptional predictions of the effective shear moduli, even up to extremely high-volume fractions.

As the rectangular fibres approach the edge of the cell clearly higher-order approximations are required. In this instance, as is shown in figure 5*a* the IEM order 6 expression is able to accurately replicate the upturn as r→1/2. Furthermore, in the limit as r→1/2 this medium becomes a layered structure. In this limit, there are exact expressions for the two shear moduli, which are [25,26]

5.3 $$\mu_{1}^{*}={\left((1-\phi )+\frac{\phi }{m}\right)}^{-1}\;\mathrm{and}\;\mu_{2}^{*}=(1-\phi )+m\phi$$

for a volume fraction *ϕ* of layered material–here this is clearly *ϕ*=1/2 and these results for $\mu_{1}^{*}$ and $\mu_{2}^{*}$ at *ϕ*=1/2 are indicated by the black squares in figure 6*a*,*b*.

**Figure 7.**
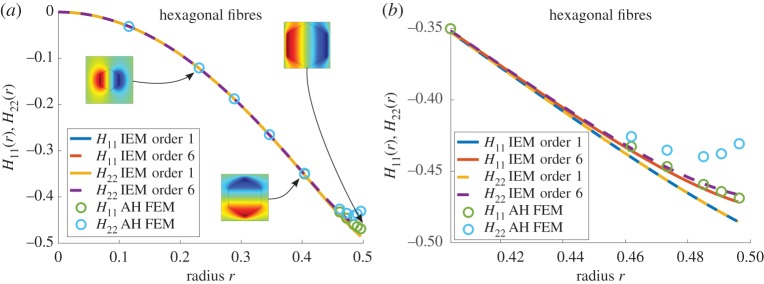
Plot of *H*_11_(*r*) and *H*_22_(*r*) for *m*=18.75 when hexagonal fibres are arranged on a square lattice, with associated solutions of the cell problem from the MAH inset (*a*) and a close-up for high radii (*b*). (Online version in colour.)

**Figure 8.**
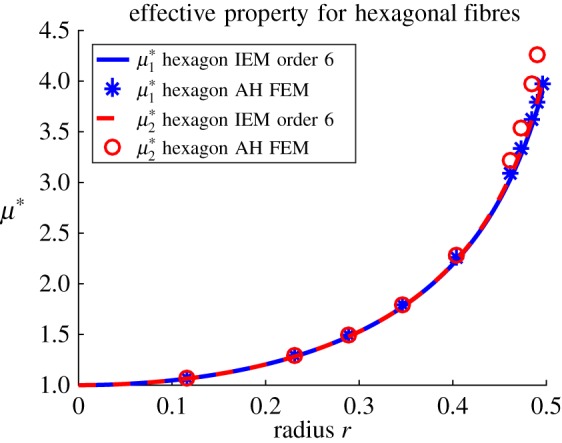
Plot of effective antiplane shear moduli $\mu_{1}^{*}$ and $\mu_{2}^{*}$ for *m*=18.75 when hexagonal-shaped fibres are arranged on a square lattice. (Online version in colour.)

Moving onto the consideration of a more complex fibre shape, figure 7 illustrates plots of the **H**-tensor in the case of a hexagonal cross section, with a magnified region of high radius plotted in figure 7*b*. The effective shear moduli are plotted in figure 8. Once again, there is extremely good agreement between the two methods and even the slight disagreement in calculations of the **H**-tensor makes little difference to the predictions of the effective moduli.

It should be noted that for a general polygonal shape (including the hexagonal case), the notion of ‘radius’, against which some results above are plotted, needs some interpretation. In fact for the case of square cells considered above, referring to (2.7) the scaled area is R=1, and referring to (2.2), the radius *r* here is chosen as $\max_{\theta \in [0,2\pi ]}\ell f(\theta )$. This ensures that *r*∈[0,0.5] for all plots.

Of great utility when describing general-shaped fibres is the so-called *superellipse* function defined as [27]

$$f(\theta )={\left({\left|\frac{1}{a}\cos\left(\frac{m}{4}\theta \right)\right|}^{n_{2}}+{\left|\frac{1}{b}\sin\left(\frac{m}{4}\theta \right)\right|}^{n_{3}}\right)}^{-1/n_{1}}.$$

In the hexagonal case above, *a* and *b* are 1, *n*_1_=100, *m*=6 and *n*_2_=*n*_3_=62. Alternatively for the rectangle, *a*=2, *b*=1, *m*=4 and *n*_1_=*n*_2_=*n*_3_=200.

## 6. Conclusion

New, explicit expressions for the effective properties of inhomogeneous media have been derived in the case of the scalar wave problem associated with periodic two-dimensional FRC in which the fibres have non-circular cross section. Results can be interpreted in the sense of low-frequency antiplane elastic waves, acoustics, etc. and are also even more broad in applicability due to the link with potential problems (table 1). The fibres in question can be of general cross section and results obtained have been validated successfully with the classical MAH. In the latter method, the cell-problem for general shapes must always be calculated numerically and the cell problem solution must be recomputed whenever parameters are altered. The advantage of the present scheme is that the resulting expressions can be used for general volume fractions without recourse to computational methods each time the volume fraction is modified. The method is designed with general fibre cross-sectional shapes in mind, but in the special case of centrally symmetric shapes, results such as (4.3) and (4.7) may be obtained. Extensions to the case of full elastodynamics and three-dimensional scenarios for both the potential problem and elastodynamics are currently underway.

## Data accessibility

This work is associated with methodology. Therefore, all research data supporting this article are directly available within this publication.

## Appendix A. Two-dimensional expansion for *J*_*δξ*_

Employ the following plane polar coordinates $\text{x}-\text{r}=\hat{\rho }(\cos\hat{\theta },\sin\hat{\theta }),\text{y}-\text{r}=\rho (\cos\theta ,\sin\theta )$ in (3.10), with $\rho ,\hat{\rho }\geq 0$ and $0\leq \theta ,\hat{\theta }<2\pi$. Also define the boundary of the cross-sectional shape as $\hat{\rho }=f(\hat{\theta })$ where $f(\hat{\theta })\geq 1$ for all $\hat{\theta }$. Therefore,

A 1 $$J_{\delta \xi }(\text{y})=\frac{1}{4\pi \delta !\xi !}\int_{V}{\hat{\rho }}^{\delta +\xi +1}\cos^{\delta }\hat{\theta }\sin^{\xi }\hat{\theta }\ln(\rho^{2}+{\hat{\rho }}^{2}-2\rho \hat{\rho }\cos(\theta -\hat{\theta }))\;d\hat{\rho }\;d\hat{\theta }.$$

In general, it is clearly not possible to obtain an analytical form for *J*_*δξ*_(**y**) and it must be evaluated numerically for a given **y**, hence the motivation for a Taylor expansion to approximate such functions. Therefore, pose expansion (3.11). In terms of the local coordinates, this may be rewritten as

A 2 $$J_{\delta \xi }(\rho ,\theta )=a_{0}^{\delta \xi }(\rho )+\sum_{n=1}^{P+2}a_{n}^{\delta \xi }(\rho )\cos(n\theta )+b_{n}^{\delta \xi }(\rho )\sin(n\theta ),$$

where the functions $a_{n}^{\delta \xi }$ and $b_{n}^{\delta \xi }$ depend on the $D_{pq}^{\delta \xi }$ coefficients from (3.11). This dependence is best found in a symbolic package such as Mathematica, so that for example

A 3 $$a_{0}^{\delta \xi }(\rho )=D_{00}^{\delta \xi }+\frac{1}{4}\rho^{2}(D_{20}^{\delta \xi }+D_{02}^{\delta \xi })+\frac{1}{64}\rho^{4}(D_{40}^{\delta \xi }+2D_{22}^{\delta \xi }+D_{04}^{\delta \xi })+\cdots .$$

Now one can employ orthogonality of trigonometric functions in order to define relationships between $D_{pq}^{\delta \xi }$. In this vein, introduce the operators $C_{m}$ and $S_{m}$ as those that multiply by $\alpha_{m}\cos(m\theta )$, *m*≥0 and $\alpha_{m}\sin(m\theta )$, *m*≥1 for integer *m* and then integrate over *θ*∈[0,2*π*] where

$$\alpha_{m}=\left\{ \begin{array}{ll} \frac{1}{2\pi }, & m=0,\\ \frac{1}{\pi }, & m\geq 1. \end{array}\right.$$

Apply these operators to the forms of *J*_*δξ*_ in (6.1) and (6.2), equate and set *ρ*=1, to obtain

A 4 $$\begin{array}{rl} a_{0}^{\delta \xi }(1) & \;=\frac{1}{8\pi^{2}\delta !\xi !}\int_{D}{\hat{\rho }}^{\delta +\xi +1}\cos^{\delta }\hat{\theta }\sin^{\xi }\hat{\theta }C_{0}(\hat{\rho },\hat{\theta })\;d\hat{\rho }\;d\hat{\theta }, \end{array}$$

A 5 $$\begin{array}{rl} a_{m}^{\delta \xi }(1) & \;=\frac{1}{4\pi^{2}\delta !\xi !}\int_{D}{\hat{\rho }}^{\delta +\xi +1}\cos^{\delta }\hat{\theta }\sin^{\xi }\hat{\theta }C_{m}(\hat{\rho },\hat{\theta })\;d\hat{\rho }\;d\hat{\theta } \end{array}$$

A 6 $$\begin{array}{rl} \mathrm{and}\;b_{m}^{\delta \xi }(1) & \;=\frac{1}{4\pi^{2}\delta !\xi !}\int_{D}{\hat{\rho }}^{\delta +\xi +1}\cos^{\delta }\hat{\theta }\sin^{\xi }\hat{\theta }S_{m}(\hat{\rho },\hat{\theta })\;d\hat{\rho }\;d\hat{\theta }, \end{array}$$

where

A 7 $$C_{m}(\hat{\rho },\hat{\theta })=\int_{0}^{2\pi }\cos(m\theta )\ln({\hat{\rho }}^{2}+1-2\hat{\rho }\cos(\theta -\hat{\theta }))\;d\theta \;$$

and

A 8 $$S_{m}(\hat{\rho },\hat{\theta })=\int_{0}^{2\pi }\sin(m\theta )\ln({\hat{\rho }}^{2}+1-2\hat{\rho }\cos(\theta -\hat{\theta }))\;d\theta .$$

Note that $f(\hat{\theta })\geq 1$. Perform the change of variables, $\psi =\theta -\hat{\theta }$, employ double angle formulae for the trigonometric functions and use the following identities taken from eqn 15 of sec.4.224 and eqn 6 of sec.4.397 of [28]:

$$\begin{array}{rl} \int_{0}^{2\pi }\ln({\hat{\rho }}^{2}+1-2\hat{\rho }\cos\psi )\;d\psi & \;=\left\{ \begin{array}{ll} 0, & {\hat{\rho }}^{2}<1,\\ 2\pi \ln{\hat{\rho }}^{2}, & {\hat{\rho }}^{2}>1. \end{array}\right.\\ \int_{0}^{2\pi }\cos(m\psi )\ln({\hat{\rho }}^{2}+1-2\hat{\rho }\cos\psi )\;d\psi & \;=\left\{ \begin{array}{ll} -\frac{2\pi }{m}{\hat{\rho }}^{m}, & {\hat{\rho }}^{2}<1,\\ -\frac{2\pi }{m}{\hat{\rho }}^{-m}, & {\hat{\rho }}^{2}>1, \end{array}\right. \end{array}$$

where *m*≥1 in the latter. One then finds that

$$\begin{array}{rl} a_{0}^{\delta \xi }(1) & \;=\frac{1}{2\pi \delta !\xi !{\left(\delta +\xi +2\right)}^{2}}\int_{0}^{2\pi }\cos^{\delta }\hat{\theta }\sin^{\xi }\hat{\theta }[{\left(f(\hat{\theta })\right)}^{\delta +\xi +2}((\delta +\xi +2)\ln f(\hat{\theta })-1)+1)]\;d\hat{\theta }\\ & \;=A_{0}^{\delta \xi }, \end{array}$$

where integration by parts was used for the *ζ* integration and a different notation has been associated with this integral form so that it may be referred to later. Also,

A 9 $$a_{m}^{\delta \xi }(1)=-\frac{1}{2\pi \delta !\xi !}\int_{0}^{2\pi }\cos^{\delta }\hat{\theta }\sin^{\xi }\hat{\theta }\cos(m\hat{\theta })\left[\frac{1}{\delta +\xi +m+2}+J_{\delta +\xi -m}(\hat{\theta })\right]d\hat{\theta }=A_{m}^{\delta \xi },$$

where

$$J_{\delta +\xi -m}(\hat{\theta })=\left\{ \begin{array}{ll} \ln(f(\hat{\theta })), & \delta +\xi -m=-2,\\ \frac{{\left(f(\hat{\theta })\right)}^{\delta +\xi -m+2}-1}{\delta +\xi -m+2}, & \text{otherwise}. \end{array}\right.$$

Similarly,

$$b_{m}^{\delta \xi }(1)=-\frac{1}{2\pi \delta !\xi !}\int_{0}^{2\pi }\cos^{\delta }\hat{\theta }\sin^{\xi }\hat{\theta }\sin(m\hat{\theta })\left[\frac{1}{\delta +\xi +m+2}+J_{\delta +\xi -m}(\hat{\theta })\right]d\hat{\theta }=B_{m}^{\delta \xi }.$$

Equating these with the relations linking $a_{n}^{\delta \xi },b_{n}^{\delta \xi }$ (evaluated at *ρ*=1) to the coefficients $D_{pq}^{\delta \xi }$, such as (6.3), one obtains a linear system in $D_{pq}^{\delta \xi }$. However, it transpires that more conditions are needed in order to close the system. These are obtained by applying the Laplacian operator to the forms of *J*_*δξ*_ in (3.10) and (3.11), equating and exploiting the fact that ∇^2^*G*_0_=*δ*(***y***−***x***), to find

$$\begin{array}{rl} \frac{{\left(y_{1}-r_{1}\right)}^{\delta }{\left(y_{2}-r_{2}\right)}^{\xi }}{\delta !\xi !} & \;=\sum_{n=2:p+q=2}^{N}\frac{D_{pq}^{\delta \xi }}{p!q!}(p(p-1){\left(y_{1}-r_{1}\right)}^{p-2}{\left(y_{2}-r_{2}\right)}^{q}\\ & \;\;+q(q-1){\left(y_{1}-r_{1}\right)}^{p}{\left(y_{2}-r_{2}\right)}^{q-2}). \end{array}$$

Comparing coefficients of (*y*_1_−*r*_1_)^*i*^(*y*_2_−*r*_2_)^*j*^ provides the further equations relating the coefficients $D_{pq}^{\delta \xi }$. Some thought leads to the conclusion that for *p*,*q*≥2

A 10 $$D_{(p+2)q}^{\delta \xi }+D_{p(q+2)}^{\delta \xi }=\delta_{p\delta }\delta_{q\xi }.$$

This additional set of equations closes the linear system.

**(a) Linear system**

The linear system consists of (A 8) and equations, such as e.g.

$$a_{0}^{\delta \xi }(1)=D_{00}^{\delta \xi }+\frac{1}{4}(D_{20}^{\delta \xi }+D_{02}^{\delta \xi })+\frac{1}{64}(D_{40}^{\delta \xi }+2D_{22}^{\delta \xi }+D_{04}^{\delta \xi })+\cdots =A_{0}^{\delta \xi }.$$

It may be written in the form

ID=F+R,

where

$$\begin{array}{rl} \text{D} & \;={\left[D_{00}\;D_{10}\;D_{01}\;D_{20}\;D_{11}\;D_{30}\;D_{21}\;D_{40}\;D_{31}\ldots \right]}^{T}\\ \text{F} & \;={\left[A_{0}\;A_{1}\;B_{1}\;A_{2}\;B_{2}\;A_{3}\;B_{3}\;A_{4}\;B_{4}\cdots \right]}^{T}, \end{array}$$

and the vector **R** is essentially a sparse vector including some constants that arise due to the Laplacian simplification on the left-hand side. The following examples are given due to their relevance to the example systems in §4.

**(i) Elliptical fibre second-order case: P=0, *δ*=*ξ*=0.**

For this case, only *J*_00_(*ρ*,*θ*) is sought as a polynomial expansion of the form

A 11 $$J_{00}={\hat{D}}_{00}^{00}+D_{10}^{00}({\tilde{y}}_{1}-{\tilde{r}}_{1})+D_{01}^{00}({\tilde{y}}_{2}-{\tilde{r}}_{2})+\frac{D_{20}^{00}}{2}{\left({\tilde{y}}_{1}-{\tilde{r}}_{1}\right)}^{2}+\frac{D_{02}^{00}}{2}{\left({\tilde{y}}_{2}-{\tilde{r}}_{2}\right)}^{2}.$$

The above expressions clarify better why P=0 is referred to as the second-order case: the sum of the lower indices of the *D* constants never exceed 2. In this case, the only non-trivial coefficients that have an influence on the shape factor term are

$$D_{20}^{00}=\frac{b}{\left(a+b\right)}\;\mathrm{and}\;D_{02}^{00}=\frac{a}{\left(a+b\right)},$$

so, using (3.12) and (3.13), the shape factor for this system has the form

A 12 $${\hat{A}}_{00}^{\left(1\right)}=-\frac{b}{\left(a+b\right)}{\hat{W}}_{00}^{\left(1\right)}\;\mathrm{and}\;{\hat{A}}_{00}^{\left(2\right)}=-\frac{a}{\left(a+b\right)}{\hat{W}}_{00}^{\left(2\right)}.$$

**(ii) Circular fibre fourth-order case example: P=2, *δ*=2,*ξ*=0**

Note that in order to fully consider equations (3.27) and (3.28) to fourth order the cases *δ*=*ξ*=1 and *δ*=0, *ξ*=2 must also be considered. The case *δ*=2, *ξ*=0 has been chosen merely as an example to illustrate how the system for the shape factor works at this order.

In this instance, the shape factor may be written as

$$\begin{array}{rl} {\hat{A}}_{20}^{\left(1\right)} & \;=\frac{1}{16}{\hat{W}}_{00}^{\left(1\right)}-\frac{1}{8}{\hat{W}}_{02}^{\left(1\right)}-\frac{7}{8}{\hat{W}}_{20}^{\left(1\right)}-\frac{1}{8}{\hat{W}}_{11}^{\left(2\right)}\\ \mathrm{and}\;{\hat{A}}_{20}^{\left(2\right)} & \;=-\frac{1}{16}{\hat{W}}_{00}^{\left(2\right)}+\frac{1}{8}{\hat{W}}_{02}^{\left(2\right)}-\frac{1}{8}{\hat{W}}_{20}^{\left(2\right)}-\frac{1}{8}{\hat{W}}_{11}^{\left(1\right)}. \end{array}$$

The fact that this shape factor depends on displacement gradient moments for *δ*=*ξ*=1 and *δ*=0, *ξ*=2 illustrates why those choices for *δ* and *ξ* must also be considered in order to fully establish this fourth-order problem.

## Appendix B. Lattice sums

Recall the definition of *L*_0_[*m*,*n*] from (3.22). While it has already been shown that this sums to zero when either *m*, *n* or both *m* and *n* are odd, and that many sums are equivalent to each other through the identity (3.26), it still remains to evaluate the non-trivial sums, when both *m* and *n* are even. Attention is restricted to parallelogram-shaped lattices, which will in turn restrict the forms of ***u*** that appear in the summation. For ease of exposition, consider the further restriction of rectangular-shaped lattices, so that summations are over integer *u*,*v* such that ***u***=(*Bu*,*v*), where *B*≥1 is the aspect ratio.

Consider the case of the lattice sum *L*_0_[2,0] (equivalent to −*L*_0_[0,2] by the identity (3.26)), which will involve the singular part of $\partial_{u_{1}}^{2}G_{0}(\text{u})$ and is

$$S_{0}[2,0]=\frac{u_{2}^{2}-u_{1}^{2}}{2\pi {\left(u_{1}^{2}+u_{2}^{2}\right)}^{2}}.$$

Employing this in the lattice sum and considering a rectangular lattice, this gives

$$L_{0}[2,0]=\frac{1}{2\pi }\sum_{\left(u,v)\neq (0,0\right)}\frac{v^{2}-B^{2}u^{2}}{{\left(B^{2}u^{2}+v^{2}\right)}^{2}}.$$

By isolating the parts of the sum where *u*=0 and *v*=0 in turn, the above may be written as

$$L_{0}[2,0]=\frac{1}{\pi }\left(\sum_{v=1}^{\infty }\frac{1}{v^{2}}-\sum_{u=1}^{\infty }\frac{1}{B^{2}u^{2}}+2\sum_{u=1}^{\infty }\sum_{v=1}^{\infty }\frac{v^{2}-B^{2}u^{2}}{{\left(B^{2}u^{2}+v^{2}\right)}^{2}}\right),$$

where sums from −∞ to ∞ excluding 0 are replaced by twice the sum from 1 to ∞ since the powers of *u* and *v* are all even. By first performing the summation with respect to *v*,

$$\begin{array}{rl} L_{0}[2,0] & \;=\frac{1}{\pi }\left(\frac{\pi^{2}}{6}-\sum_{u=1}^{\infty }\frac{1}{B^{2}u^{2}}+2\sum_{u=1}^{\infty }\left[\frac{1}{2B^{2}u^{2}}-\frac{\pi^{2}}{2}{\text{cosech}}^{2}(\pi Bu)\right]\right)\\ & \;=\frac{\pi }{6}-\pi \sum_{u=1}^{\infty }{\text{cosech}}^{2}(\pi Bu). \end{array}$$

Note that in the special case of square lattices, *B*=1, performing the summation with respect to *u* yields *L*_0_[2,0]=1/2. It transpires that in the case of square lattices, even more of the lattice sums become trivial, since

B 1 $$L_{0}[2(2i+1),0]=0\;\forall \;i\geq 1.$$

Thus via the identity (3.26), *L*_0_[*m*,*n*] will be zero for any even *m* and *n* whose sum is an odd number multiplied by 2.

As a further example, in the general rectangular lattice case, *L*_0_[6,0] has the form

$$\begin{array}{rl} L_{0}[6,0] & \;=\pi^{5}\left(\frac{8}{63}-\sum_{u=1}^{\infty }[{\text{cosech}}^{2}(\pi Bu)(2\coth^{4}(\pi Bu)+11\coth^{2}(\pi Bu){\text{cosech}}^{2}(\pi Bu)+2{\text{cosech}}^{4}(\pi Bu))]\right). \end{array}$$

When *B*=1 here, it transpires that the summation term equals 8/62 and thus *L*_0_[6,0]=0 for a square lattice, which is consistent with the results derived above. Additionally, in the case of square lattices, for example

$$L_{0}[4,0]≃-3.00919,\;L_{0}[8,0]≃-3413.73\;\mathrm{and}\;L_{0}[12,0]≃-1.25117\times 10^{7}.$$

## Appendix C. Justifying the form of expansion for centrally symmetric shapes

Recall that central symmetry of the inclusion shape is defined such that *f*(*θ*+*π*)=*f*(*θ*). First, consider the summation terms on the left sides of (3.27) and (3.28). When *δ*+*ξ* is even, including the case *δ*+*ξ*=0 of course, displacement gradient moments of odd order could appear in these terms, but only when *α*+*β* is even. This can be observed by examining the coefficient ${\hat{C}}_{\delta \xi \alpha \beta }$ as defined in (3.7), dividing the range of integration into two parts–one for $\hat{\theta }\in [0,\pi )$ and the other for $\hat{\theta }\in [\pi ,2\pi )$, and using the substitution $\hat{\theta }=\psi +\pi$ in the integral from *π* to 2*π*. By making use of trigonometric sum identities and the fact that *f*(*ψ*+*π*)=*f*(*ψ*) due to central symmetry, ${\hat{C}}_{\delta \xi \alpha \beta }$ can be transformed into an integral multiplied by the factor 1+(−1)^*δ*+*ξ*+*α*+*β*^. Therefore, with *δ*+*ξ* being even, (−1)^*δ*+*ξ*+*α*+*β*^=−1 in the case when *α*+*β* is odd and so ${\hat{C}}_{\delta \xi \alpha \beta }=0$. Then, for centrally symmetric fibre cross sections with *δ*+*ξ* being even, only terms within the quadruple sum where *α*+*β* is even will be potentially non-trivial.

With this established, notice next that each moment in the quadruple sum term is multiplied by a lattice sum. It transpires that for every combination of *α*, *β*, *i* and *j* where *i*+*j* is odd and *α*+*β* is even, at least one of the inputs of the lattice sum multiplying $W_{ij}^{\left(k\right)}$ is odd. All such scenarios are outlined in table 2. As established earlier, the lattice sum evaluates to zero if either of its inputs is odd. Therefore, no moments of odd order will appear in the quadruples sum terms in even-ordered equations (i.e. when *δ*+*ξ* is even).

**Table 2.** List of possible scenarios where both *α*+*β* is even and *i*+*j* is odd and how each scenario causes the lattice sum terms in (3.27) and (3.28) to be trivial.

*α*	*β*	*i*	*j*	reason lattice sums are trivial
even	even	odd	even	*i*+*α*, *i*+*α*+2 and *j*+*β*+1 are all odd
odd	odd	even	odd	*i*+*α*, *i*+*α*+2 and *j*+*β*+1 are all odd
even	even	even	odd	*i*+*α*+1, *j*+*β* and *j*+*β*+2 are all odd
odd	odd	odd	even	*i*+*α*+1, *j*+*β* and *j*+*β*+2 are all odd

This leaves the displacement gradient moment expansion of the shape factor (3.12) and (3.13) as the only remaining terms that can potentially involve moments of odd order. Note from the form of its expansion that the shape factor term will not include any such moments if the $D_{ij}^{\delta \xi }$ coefficients are zero when *i*+*j* is odd (hereon in described as coefficients of odd order). It transpires from the process discussed in appendix A that the coefficients of odd order and those of even order separate into two subsystems. Therefore, if all coefficients of odd order are to be zero, the subsystem of such coefficients should be homogeneous—i.e. no terms independent of the coefficients with indices totalling an odd number should be present in the subsystem.

Firstly note that since *δ*+*ξ* is even, the only inhomogeneous Laplace type condition that one obtains from (A 8) is guaranteed not to involve any odd-ordered coefficients. Therefore, it just remains to be shown that the equations in the system from appendix A related to odd-ordered coefficients are homogeneous. This requires the $A_{m}^{\delta \xi }$ and $B_{m}^{\delta \xi }$ terms to be zero when *m* is odd. When *m* is odd these reduce to

$$\begin{array}{rl} {\hat{A}}_{m}^{\delta \xi } & \;=\int_{0}^{2\pi }\cos^{\delta }\hat{\theta }\sin^{\xi }\hat{\theta }\cos m\hat{\theta }f^{\left(\delta +\xi +2-m\right)}(\hat{\theta })\;d\hat{\theta }\\ \mathrm{and}\;{\hat{B}}_{m}^{\delta \xi } & \;=\int_{0}^{2\pi }\cos^{\delta }\hat{\theta }\sin^{\xi }\hat{\theta }\sin m\hat{\theta }f^{\left(\delta +\xi +2-m\right)}(\hat{\theta })\;d\hat{\theta }, \end{array}$$

where

$${\hat{A}}_{m}^{\delta \xi }=-2m\pi \delta !\xi !(\delta +\xi +2-m)A_{m}^{\delta \xi }\;\mathrm{and}\;{\hat{B}}_{m}^{\delta \xi }=-2m\pi \delta !\xi !(\delta +\xi +2-m)B_{m}^{\delta \xi }.$$

As was the case when examining ${\hat{C}}_{\delta \xi \alpha \beta }$, splitting the range of integration into two parts—one for $\hat{\theta }\in [0,\pi )$ and the other for $\hat{\theta }\in [\pi ,2\pi )$ then substituting $\hat{\theta }=\psi +\pi$ into the latter and making further use of the central symmetry of *f* and using trigonometric sum identities, the integral forms for ${\hat{A}}_{m}^{\delta \xi }$ and ${\hat{B}}_{m}^{\delta \xi }$ can be rewritten as single integrals multiplied by the term (1+(−1)^*m*+*δ*+*ξ*^). Since *m* is odd and *δ*+*ξ* is even, 1+(−1)^*m*+*δ*+*ξ*^=0 and therefore $A_{m}^{\delta \xi }$ and $B_{m}^{\delta \xi }$ are zero. Therefore, any subsystem of equations in coefficients $D_{ij}^{\delta \xi }$ where *i*+*j* is odd will be homogeneous in these coefficients and hence the solution for this subset of coefficients will be trivial. This means that, when *δ*+*ξ* is even, the expansion of *J*_*δξ*_(***y***) will not feature any powers of the coordinate basis of odd order.

With regard to the shape factor therefore, the above results in no displacement gradient moments of odd order appearing in (3.12) and (3.13) when *δ*+*ξ* is even and the fibre cross-sectional shape is centrally symmetric. Therefore, in this case, no fractional powers of the volume fraction *ϕ* shall appear in the system, which means that asymptotically expanding the moments and *g*(*γ*_1_) in powers of *ϕ*^1/2^ is unnecessary. Instead, one can expand in powers of *ϕ*.

## References

[1] Parnell WJ, Abrahams ID 2006 Dynamic homogenization in periodic fibre reinforced media. Quasi-static limit for SH waves. **Wave Motion** 43, 474–498. (doi:10.1016/j.wavemoti.2006.03.003)

[2] Milton GW 2001 *The theory of composites*, 1st edn Cambridge, UK: Cambridge University Press.

[3] Bakhvalov N, Panasenko G 1989 *Homogenisation: averaging processes in periodic media: mathematical problems in the mechanics of composite materials*. Berlin, Germany: Springer.

[4] Edelstein AS, Cammarata RC (eds) 1996 *Nanomaterials: synthesis, properties and applications*, 2nd edn New York, NY: CRC Press.

[5] Bensoussan A, Lions J-L, Papanicolaou G 1978 *Asymptotic analysis for periodic structures*. Amsterdam, The Netherlands: North-Holland Publishing Company.

[6] Sabina FJ, Bravo-Castillero J, Guinovart-Díaz R, Rodríguez-Ramos R, Valdiviezo-Mijangos OC 2002 Overall behaviour of two-dimensional periodic composites. *Int. J. Solids Struct.* 39, 483–497. (doi:10.1016/S0020-7683(01)00107-X)

[7] Parnell WJ, Abrahams ID 2008 Homogenization for wave propagation in periodic fibre-reinforced media with complex microstructure I-Theory. *J. Mech. Phys. Soilds* 56, 2521–2540. (doi:10.1016/j.jmps.2008.02.003)

[8] Iwakuma T, Nemat-Nasser S 1983 Composites with periodic microstructure. *Comp. Struct.* 16, 13–19. (doi:10.1016/0045-7949(83)90142-6)

[9] Nemat-Nasser S, Hori M 1999 *Micromechanics: overall properties of heterogeneous materials*. Amsterdam, The Netherlands: North-Holland.

[10] Helsing J 1995 An integral equation method for elastostatic of periodic composites. *J. Mech. Phys. Solids* 43, 815–828. (doi:10.1016/0022-5096(95)00018-E)

[11] Moulinec H, Suquet P 1994 A fast numerical method for computing the linear and nonlinear properties of composites. *C. R. Acad. Sci. II* 318, 1417–1423.

[12] Michel J, Moulinec H, Suquet P 1999 Effective properties of composite materials with periodic microstructure: a computational approach. *Comput. Methods Appl. Mech. Eng.* 172, 109–143. (doi:10.1016/S0045-7825(98)00227-8)

[13] Bonnet G 2007 Effective properties of elastic periodic composite media with fibers. *J. Mech. Phys. Solids* 55, 881–899. (doi:10.1016/j.jmps.2006.11.007)

[14] Kushwaha MS, Halevi P, Martínez G, Dobrzynski L, Djafari-Rouhani B 1993 Acoustic band structure of periodic elastic composites. *Phys. Rev. Lett.* 71, 2022 (doi:10.1103/PhysRevLett.71.2022) 10054563 10.1103/PhysRevLett.71.2022

[15] Zalipaev VV, Movchan AB, Poulton CG, McPhedran RC 2002 Elastic waves and homogenization in oblique periodic structures. *Proc. R. Soc. Lond. A* 458, 1887–1912. (doi:10.1098/rspa.2001.0948)

[16] Craster RV, Kaplunov J, Pichugin AV 2010 High-frequency homogenization for periodic media. *Proc. R. Soc. A* 466, 2341–2362. (doi:10.1098/rspa.2009.0612)

[17] Hussein MI, Leamy MJ, Ruzzene M 2014 Dynamics of phononic materials and structures: historical origins, recent progress and future outlook. *Appl. Mech. Rev.* 66, 040802 (doi:10.1115/1.4026911)

[18] Parnell WJ, Abrahams ID 2008 A new integral equation approach to elastodynamic homogenization. *Proc. R. Soc. A* 464, 1461–1482. (doi:10.1098/rspa.2007.0254)

[19] Brazier-Smith PR 2002 A unified model for the properties of composite materials. In *Proc. IUTAM Symp. on scattering and diffraction in fluid mechanics and elasticity, Manchester, UK, 16–20 July 2000*. Dordrecht, The Netherlands: Kluwer.

[20] Parnell WJ, Abrahams ID, Brazier-Smith PR 2010 Effective properties of a composite half-space: exploring the relationship between homogenization and multiple-scattering theories. *Q. J. Mech. Appl. Math.* 63, 145–175. (doi:10.1093/qjmam/hbq002)

[21] McIver R, Krynkin A 2009 Approximations to wave propagation through a lattice of Dirichlet scatterers. *Waves Random Complex Media* 19, 347–365. (doi:10.1080/17455030802616855)

[22] Guo S, McIver P 2011 Propagation of elastic waves through a lattice of cylindrical cavities. *Proc. R. Soc. A* 467, 2962–2982. (doi:10.1098/rspa.2011.0069)

[23] Mura T 1982 *Micromechanics of defects in solids*. Dordrecht, The Netherlands: Kluwer.

[24] Parnell WJ 2016 The Eshelby, Hill, Moment and Concentration tensors for ellipsoidal inhomogeneities in the Newtonian potential problem and linear elastostatics. *J. Elast.* 125, 231–294. (doi:10.1007/s10659-016-9573-6)

[25] Backus G 1962 Long-wave elastic anisotropy produced by horizontal layering. *J. Geophys. Res.* 67, 4427–4440. (doi:10.1029/JZ067i011p04427)

[26] Parnell WJ, Calvo-Jurado C 2015 On the computation of the Hashin–Shtrikman bounds for transversely isotropic two-phase linear elastic fibre-reinforced composites. *J. Eng. Math.* 95, 295–323. (doi:10.1007/s10665-014-9777-3)

[27] Gielis J 2003 A generic geometric transformation that unifies a wide range of natural and abstract shapes. *Am. J. Bot.* 90, 333–338. (doi:10.3732/ajb.90.3.333) 21659124 10.3732/ajb.90.3.333

[28] Gradshteyn IS, Ryzhik IM 2014 Definite integrals of elementary functions. In *Table of integrals, series, and products* (ed. D Zwillinger), 8th edn, ch. 3–4. Burlington, MA: Academic Press.

